# Cross-Format Integration of Auditory Number Words and Visual-Arabic Digits: An ERP Study

**DOI:** 10.3389/fpsyg.2021.765709

**Published:** 2021-11-23

**Authors:** Sabrina Finke, Ferenc Kemény, Francina J. Clayton, Chiara Banfi, Anna F. Steiner, Corinna M. Perchtold-Stefan, Ilona Papousek, Silke M. Göbel, Karin Landerl

**Affiliations:** ^1^Institute of Psychology, University of Graz, Graz, Austria; ^2^Institute of Education and Psychology at Szombathely, Eötvös Loránd University, Budapest, Hungary; ^3^Department of Psychology, University of York, York, United Kingdom; ^4^Institute for Medical Informatics, Statistics and Documentation, Medical University of Graz, Graz, Austria; ^5^FH JOANNEUM, University of Applied Sciences, Graz, Austria; ^6^Department of Special Needs Education, University of Oslo, Oslo, Norway; ^7^BioTechMed-Graz, Graz, Austria; ^8^Department of Cognitive Science, Macquarie University, Sydney, NSW, Australia

**Keywords:** ERP, numerical cognition, cross-format integration, symbolic numbers, N1, N400

## Abstract

Converting visual-Arabic digits to auditory number words and vice versa is seemingly effortless for adults. However, it is still unclear whether this process takes place automatically and whether accessing the underlying magnitude representation is necessary during this process. In two event-related potential (ERP) experiments, adults were presented with identical (e.g., “one” and 1) or non-identical (e.g., “one” and 9) number pairs, either unimodally (two visual-Arabic digits) or cross-format (an auditory number word and a visual-Arabic digit). In Experiment 1 (N=17), active task demands required numerical judgments, whereas this was not the case in Experiment 2 (N=19). We found pronounced early ERP markers of numerical identity unimodally in both experiments. In the cross-format conditions, however, we only observed late neural correlates of identity and only if the task required semantic number processing (Experiment 1). These findings suggest that unimodal pairs of digits are automatically integrated, whereas cross-format integration of numerical information occurs more slowly and involves semantic access.

## Introduction

Whether number words and visual-Arabic digits are automatically and involuntarily linked is an enduring open question in cognitive psychology. Every day, we navigate through our modern literate world by integrating numerical information from different sensory modalities: Following the station announcement that our train leaves from platform five, we search for the corresponding visual-Arabic digit 5. In adults, switching from one number format to another seems to happen effortlessly. While this daily experience suggests an automatic integration of number words and digits, empirical evidence is still critically lacking. Therefore, the current paper addresses the question whether the mental representation of a specific digit is automatically and involuntarily activated upon hearing the corresponding number word. Here, we attempt to unravel the neurocognitive mechanisms underlying this integration process.

Already at a young age children acquire the skill to link verbal numbers to their written digit counterparts: Words for small magnitudes (e.g., “two dogs,” “three little pigs”) are among the first words children learn (Durkin et al., 1986), and there is evidence suggesting that some children understand the meaning of single visual-Arabic digits as early as 18months (Mix, 2009). When children enter school, their production and comprehension of single visual-Arabic digits are already near perfect (Moura et al., 2015). Following many years of repeated exposure to numbers and frequent experience translating between different number formats, it seems intuitive that visual and verbal representations become increasingly linked. Representations of number words and digits may overlap to such an extent that one representation automatically activates the other. The strength of this link between number words and digits might be related to arithmetic performance in different age groups (children: Göbel et al., 2014; Malone et al., 2020; adults: Sasanguie and Reynvoet, 2014 but see Lyons et al., 2014; Sasanguie et al., 2017; Lin and Göbel, 2019). It has even been put forward that the mapping between number words and Arabic numbers might act as a “gatekeeper (or barrier) in the development of formal mathematical knowledge” (Purpura et al., 2013, p. 460).

The existence of a direct and automatic association of number words and digits was already proposed in the earliest version of the Triple Code Model (Dehaene and Cohen, 1995). According to this model, numbers are processed in three different numerical codes: The visual-Arabic number form processes numbers represented as digits, while spoken or written number words are represented in the verbal word frame. According to the model, both visual-Arabic digits and number words are symbols that do not *per se* contain any semantic information. Number semantics are only represented by the analogue magnitude representation, which is involved in all processes accessing the non-symbolic quantity of a given number. Bidirectional translational paths are postulated to directly link the different numerical codes. Crucially, the Triple Code Model includes an asemantic transcoding route between representations of visual-Arabic digits and number words that has been shown to rely on left hemispheric pathways (Dehaene and Cohen, 1995). In other words, there is evidence for a direct route between the symbolic numerical representations of number words and of visual-Arabic digits, without the use of an indirect route through the activation of the underlying magnitude representation. In contrast, semantic models propose that number words and digits are only indirectly linked *via* their underlying meaning in terms of numerical magnitude. Specifically, semantic models of transcoding (e.g., Power and Dal Martello, 1990; McCloskey, 1992) assume that the source number is first transformed into an abstract analogue magnitude, which in turn then is transformed into the target number.

However, we frequently employ symbolic numbers in the absence of any actual numerical meaning. The magnitudes underlying certain combinations of digits, for example post codes, PIN codes, and telephone country codes do not necessarily carry relevant magnitude information.^1^ The view that number words and digits are linked directly without an intermediary step of access to number semantics is supported by asemantic transcoding models (e.g., Power and Dal Martello, 1997; Barrouillet et al., 2004; Dotan and Friedmann, 2018). For example, the ADAPT model (Barrouillet et al., 2004) suggests that a verbal number word is parsed until a single word unit is identified. Each word unit or chunk either corresponds to lexicalized or non-lexicalized elements. Lexicalized elements can be directly retrieved from long-term memory and consist of lexical primitives including single-digit numbers one to nine, as well as teens, decades, and separators, such as hundred and thousand. On the other hand, non-lexicalized elements (e.g., “238”) require complementary procedures. These can be best described as an algorithmic transcoding strategy which serves as a back-up if direct memory retrieval fails. Critically though, neither lexicalized nor non-lexicalized elements require any access to the underlying number semantics during the entire process of linking number words and their corresponding visual-Arabic digits.

Researchers have argued for the existence of a direct link between number words and digits based on behavioral findings from number comparison and matching tasks (Lyons et al., 2012; Marinova et al., 2018). Lyons et al. (2012) instructed participants to indicate the larger of a pair of quantities, presented in a single format (visual-Arabic digits, written number words or dot arrays), in a mixed symbolic format (visual-Arabic digits and written number words), or in a mixed symbolic-non-symbolic format (visual-Arabic digits and dot arrays). Participants showed switch costs in terms of significantly longer response times for mixed non-symbolic-symbolic pairs compared to single-format pairs. However, no switch costs were observed when comparing mixed symbolic pairs with single-format symbolic pairs, suggesting that number words and digits are closely linked. Marinova et al. (2018) were able to extend these findings using a task that did not require explicit magnitude judgments: They showed that also in a number matching task in which participants had to judge whether two quantities were numerically identical, participants were slower to compare mixed non-symbolic and symbolic pairs (tone sequences and digits) than their mixed symbolic counterparts (auditory number words and visual digits).

The fact that the co-activation of purely symbolic representations was faster than the co-activation of symbolic and non-symbolic representations points to a direct link between the visual-Arabic number form and the verbal word frame. However, task demands in number matching tasks may also elicit an activation of semantic content, that is, the numerical value of abstract number symbols. Therefore, findings from number matching tasks only provide indirect and incomplete evidence for the direct link between number words and digits.

Neuroscientific studies offer another window into investigating the association between number words and digits. More specifically, using event-related potential (ERP) methodology allows us to examine the time course of cross-format integration. Although ERP evidence about the direct link between number words and digits is still critically lacking, previous studies identified the importance of the N1 and N400 components in the processing of numerical stimuli.

The parietal N1 component was reported to be sensitive to numerical distance (Temple and Posner, 1998) and numerical identity (Liu et al., 2018). Specifically, small numerical distances elicited larger N1 amplitudes than larger numerical distances in number comparison tasks with dots and digits in both adults and children between 5 and 9years (Temple and Posner, 1998). However, the finding of an N1 amplitude modulation by numerical distance was not consistent across studies, as others could not replicate this finding, neither for non-symbolic nor symbolic numbers (Libertus et al., 2007; Hyde and Spelke, 2009). Generally, an N1 component can provide evidence for automatic and asemantic processing.

Semantic processing of numerical information is thought to be reflected by the N400 ERP component (Niedeggen et al., 1999; Galfano et al., 2004; Szücs and Csépe, 2005; Paulsen and Neville, 2008; Szücs and Soltész, 2010; Pinhas et al., 2014). The N400 is a central negative component peaking at around 400ms, generally known to be sensitive to semantic mismatch or unexpectedness (Kutas and Federmeier, 2011). In the domain of numerical processing, the N400 was found to be sensitive to numerical identity using non-symbolic paradigms (Paulsen and Neville, 2008)^2^ and also cross-format number pairs: In a passive paradigm not requiring numerical judgments, children showed more negative N400 components to mismatch between visually presented analogous magnitudes and auditorily presented number words already at the age of 3 to 5years (Pinhas et al., 2014). Interestingly though, the authors reported that this effect of numerical identity was only observable for children who were already able to count. Overall, this suggests that once children have acquired basic counting knowledge, they automatically activate information about non-symbolic quantities and verbal number words *via* the underlying number semantic, even if they are not actively required to do so.

It is important to note that the numerical N400 effect can be dissociated from earlier N2b effects of perceptual non-match: Increased negative amplitudes have been reported at central electrode sites around 300–400ms for incorrect versus correct calculations in arithmetic verification (Niedeggen et al., 1999; Szücs and Soltész, 2010) as well as in implicit probe tasks (Galfano et al., 2009). In terms of polarity and topography, this numerical N400 effect is highly similar to the classical N400 effect, often considered to reflect Lexico-semantic processing (Szücs et al., 2007; Szücs and Soltész, 2010). In terms of timing, however, the peak of the numerical N400 typically occurs around 100ms earlier than for linguistic stimuli (Bassok et al., 2009; Guthormsen et al., 2017).

In the present study, these well-documented ERP components of numerical processing were used to investigate the temporal characteristics of unimodal and cross-format processing of symbolic number representations. In particular, we set out to test whether visual-Arabic digits and auditory number words are directly linked without explicit magnitude judgments being required as proposed by the Triple Code Model (Dehaene and Cohen, 1995) and asemantic transcoding models (Power and Dal Martello, 1997; Barrouillet et al., 2004; Dotan and Friedmann, 2018). We employed an ERP paradigm to investigate the possibly automatic link between number words and digits, both with a unimodal visual and a cross-format auditory–visual condition. If a direct link between number words and digits exists, we expect ERP effects of numerical identity to be present in both unimodal and cross-format conditions. Considering the previous literature (e.g., Liu et al., 2018), we hypothesized to find a larger N1 component for numerically identical trials than for numerically non-identical trials at parietal electrode sites. We conducted two experiments varying the access to the underlying number semantics: Experiment 1 involved numerical decisions and thus required participants to access the underlying number semantics in order to solve the active task. Experiment 2 did not involve any numerical judgments, and thus, no semantic access was needed. Due to the involvement of number semantics, we predicted an N400 effect of numerical identity with more negative deflections for non-identical than identical number pairs for Experiment 1. Observing a similar N400 effect also in Experiment 2 would suggest that pairs of numbers are linked semantically even when no access to the number semantic is required. In summary, we investigated ERP effects in response to the integration of auditory number words and visual-Arabic digits. We contrasted ERP effects of numerical identity robustly associated with automatic processing (N1 component) and semantic processing (N400 component).

## Experiment 1

### Participants

The sample comprised 17 healthy volunteers recruited at the University of Graz, Austria (age: *M*=22.6years, *SD*=2.4; 8 males and 9 females). They were all native speakers of German and had normal or corrected-to-normal vision, as well as normal hearing status. Initially, three more participants took part but had to be excluded from data analysis because of technical issues with EEG recording or because of noisy data. Psychology students received course credit for participation. The study was conducted in accordance with the Declaration of Helsinki, and ethical clearance was obtained from the ethics committee of the University of Graz. Participants provided written informed consent prior to participation.

We conducted a *post hoc* sensitivity analysis with the “pwr” package (Champely, 2020) in R (R Core Team, 2020). Note that there were no estimates of effect sizes available in the literature, as the present research question had not been investigated previously. Our sensitivity analysis revealed that we would have been able to detect an effect of $\eta_{p}^{2}=0.15$ at an *α* of 0.05 and power set at 0.80 with the present sample size. As shown in the results section, the effects of numerical identity we observed for both components were even larger, which supports the adequacy of the current sample size.

### Stimuli and Procedure

Participants were presented with pairs of numbers from 1 to 9 representing either the same or different numerosities. They were asked to indicate *via* keypress whether the second number of a pair was larger or smaller than five. The paradigm consisted of a unimodal and a cross-format block. In the unimodal block, the number pairs consisted of two visual-Arabic digits. In the cross-format block, the first number of a pair was a spoken number word, whereas the second number was a visual-Arabic digit. Each block was preceded by four practice trials to ensure that participants understood the task.

In each of the two blocks, participants were exposed to 240 number pairs appearing in a pseudorandom order. Number pairs contained digits and number words corresponding to the numerosities 1, 2, 4, 6, 8, and 9. As “seven” is a disyllabic number word in German, this numerosity was not included. To obtain an identical number of numerosities below and above five, we also decided not to include the numerosity 3. Visual stimuli, that is, Arabic digits, were presented in white on a black background with a height of 3 degrees of visual angle. In 120 trials per block, the number pairs were numerically identical, meaning that both numbers of a pair corresponded to the same numerosity. In the other 120 trials of a block, the number pairs were numerically non-identical. The numerical distance between the non-identical number pairs was either small (numerical distance of 1–3) or large (numerical distance of 5–8). Each block contained 60 number pairs with small and large numerical distances, respectively. In order to avoid low-level perceptual adaptation effects, we displayed visual-Arabic digits in one of four different spatial locations at 1 degree from the center of the display, using one of four different fonts (similar to an fMRI study by Vogel et al. (2017): Arial, Calibri, Century, Times New Roman). We ensured that in each trial, both constituents of a digit pair differed in terms of spatial locations and fonts. Number words were each presented by one of four speakers (two male and two female voices).

**Figure 1:**
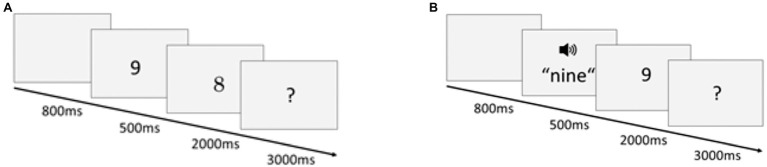
Schematic time course of **(A)** a unimodal trial with a numerically non-identical visually presented number pair and **(B)** a cross-format trial with a numerically identical auditory–visual number pair.

As shown in Figure 1, each trial began with a blank screen, displayed for 800ms. Then, the first number was presented for 500ms, before the second number appeared. This period was determined by the fact that this was the shortest possible period to present spoken number words comprehensively. Participants were asked to press the up arrow (right index finger) if the second number was larger than five or the down arrow (left index finger) if it was smaller than five. The next trial began as soon as a response was registered. If no response occurred within 2000ms after stimulus onset, a question mark was displayed for another 3,000ms. If participants did not respond within 5s of stimulus onset, the next trial was presented.

### ERP Recording and Data Analysis

Participants were seated in an acoustically and electrically shielded booth, 74cm from the center of a 1920×1080 screen (refresh rate of 144Hz). The paradigm was programmed with PsychoPy, version 1.90.1 (Peirce, 2009). Auditory stimuli were played by standard PC speakers. EEG was recorded from 19 Brain Products^™^ actiCAP active electrodes, positioned to the international 10–20 system using a BrainVision actiCHamp Research Amplifier (Brain Products^™^) with a sampling rate of 1,000Hz and a stretchable electrode cap, referenced to the nose, and re-referenced offline to a mathematically averaged ears reference (Hagemann, 2004). We measured vertical and horizontal electrooculograms (EOGs) with two bipolar channels. Electrode impedances were below 30kΩ for all electrodes. The continuous EEG was filtered (low cutoff: 0.1Hz, time constant: 15.91, 24dB/Oct; high cutoff: 100Hz, 24dB/Oct; notch filter: 50Hz). EOG artifacts were removed by automatic ocular correction, using an ICA algorithm as implemented in BrainVision Analyzer 2.1 (slope mean, over the whole data, ICA with infomax algorithm, total squared correlations to delete: 30%; Gratton et al., 1983). Other artifacts were excluded automatically (gradient criteria: more than 50μV difference between two successive data points or more than 200μV difference in a 200ms window; low activity criterion: less than 0.5μV activity in a 100ms window). The data were segmented into epochs of 700ms before onset of the second number of a pair to the end of the trial (1,000ms after stimulus onset). Because the first number of a pair was presented 500ms before the second number, the time window of −700 to −500ms served as the basis for baseline correction. Only segments with a correct response in a time window from 200 to 2000 ms were considered. All participants had at least 98 valid segments in each of the four conditions. On average, 112.82 (*SD*=4.22) numerically identical segments and 113.29 (*SD*=4.84) non-identical segments were retained for the unimodal conditions. For the cross-format conditions, an average of 114.94 (*SD*=4.98) numerically identical and 113.53 (*SD*=5.48) non-identical segments were included.

For the analysis of the N1 component, based on a previous ERP study of numerical identity (Liu et al., 2018), we averaged across a parietal electrode group including electrodes over left and right hemispheres (P3, P4, Pz, P7 and P8). The respective time window was identified as between 100 and 200ms after stimulus onset. For the analysis of the N400 component, based on previous numerical processing ERP studies (Szücs and Csépe, 2005; Avancini et al., 2014), we considered a central electrode cluster (C3, C4, Cz). For the N400 component, we identified a time window from 250 to 400ms after stimulus onset. For the N1 and N400 components, the peak was determined by detecting the most negative amplitude in the given time window for each electrode, and peak amplitude was defined as the average amplitude at peak and +/− 10ms around the peak.

All statistical analyses were carried out with numerical identity (non-identical versus identical) and modality (unimodal versus cross-format) as within-subject variables. In all ANOVAs, we conducted separate follow-up analyses for each modality condition, even in the absence of a significant interaction to confirm that the main effects were not driven by only one of the modality conditions, but were reliable in both.

In principle, numerical distance effects can be used to test semantic access, more specifically by contrasting ERP effects for small and large numerical distances. However, this is not possible in the current design, because numerical distance was confounded with response selection: As the active task required participants to judge whether the second number of the pair was larger or smaller than five, number pairs with large numerical distance were always incongruent in terms of response selection. In other words, for number pairs with a large numerical distance, one number was always smaller than five, while the other was always larger than five. For number pairs with a small numerical distance, both numbers of a pair were often congruent in terms of response selection (both numbers either smaller or larger than five) – although this was not true for all cases (e.g., number pair “4” and “6”). Due to these confounds, we did not investigate numerical distance.

### Results

The data collected for this study are publicly available on the Open Science Framework and can be accessed at https://osf.io/p7ksn/

#### Behavioral Measures: Accuracy and Reaction Times for the Numerical Decision Task

**Figure 2:**
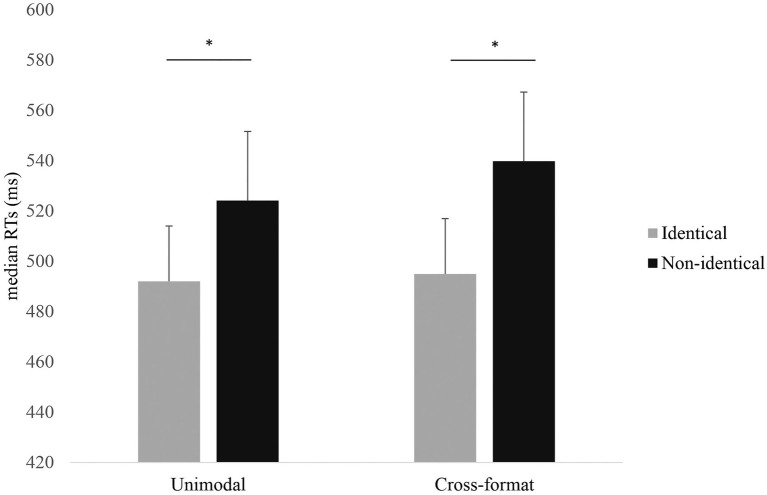
Median reaction times (RTs) by numerical identity and modality. Error bars indicate SEM. Asterisks indicate significant differences (*p*<0.05).

In a first step, we investigated participants’ behavioral performance on our novel experimental task. As expected for this simple task format, accuracy was above 94% for both the unimodal and cross-modal conditions of the numerical decision task and was not further analyzed. Only RTs for correct responses were considered for analyses. We calculated median RTs for numerically identical as well as numerically non-identical number pairs separately for the unimodal and cross-format blocks for all participants. RTs by numerical identity and experimental block are provided in Figure 2.

We conducted a two-way repeated measures ANOVA with the within-subject factors identity (identical versus non-identical number pairs) and modality (unimodal versus cross-format). The ANOVA revealed a significant main effect of identity, *F*(1,16)=35.95, *p*<0.001, $\eta_{p}^{2}$ =0.69, with higher RTs for non-identical versus identical number pairs. Neither the main effect of modality nor the identity x modality interaction were significant (both *p*s>0.341). As described above, we ran separate repeated measures ANOVAs for each modality with identity as within-subject variable. A significant effect of identity was confirmed for both the unimodal, *F*(1,17)=14.06, *p*=0.002, $\eta_{p}^{2}$ =0.47, and the cross-format condition, *F*(1,17)=21.68, *p*<0.001, $\eta_{p}^{2}$ =0.58.

#### N1

**Figure 3:**
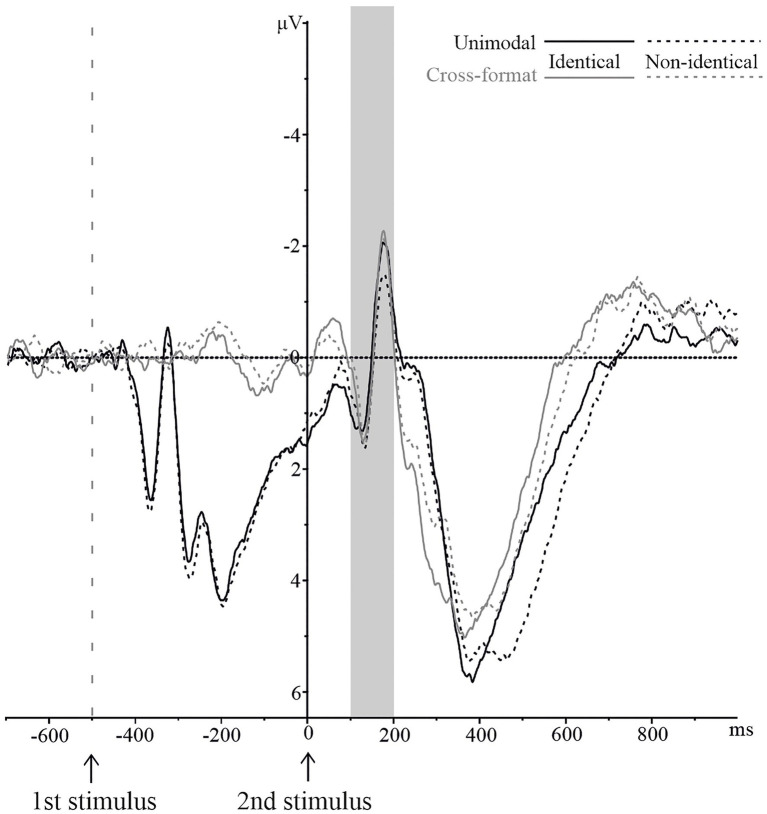
N1 component on the pooled parietal electrode cluster for numerically identical and non-identical number pairs in unimodal visual and cross-format auditory–visual blocks. Solid lines represent ERPs for identical, and dashed lines for non-identical number pairs. ERPs are shown in black for unimodal items and in grey for cross-format items.

The averaged waveforms of the parietal electrode cluster by identity and modality are depicted in Figure 3. An identity x modality ANOVA revealed a significant main effect of identity, *F*(1,16)=5.10, *p*=0.038, $\eta_{p}^{2}$ =0.24, with more negative peak amplitudes for identical than non-identical number pairs. The interaction was also significant, *F*(1,16)=4.58, *p*=0.048, $\eta_{p}^{2}$ =0.22. The main effect of modality was not significant (*F*<1).

To further analyze the identity x modality interaction, a separate repeated measures ANOVA was conducted for each modality with identity (identical vs. non-identical) as within-subject variable. The ANOVAs revealed a significant effect for the unimodal block, *F*(1,16)=8.62, *p*=0.010, $\eta_{p}^{2}$ =0.35, with more negative peak amplitudes for identical than non-identical number pairs. For the cross-format block, however, the difference between identical and non-identical pairs was not significant, *F*(1,16)=0.02, *p*=0.879, $\eta_{p}^{2}$ =0.00.

#### N400

**Figure 4:**
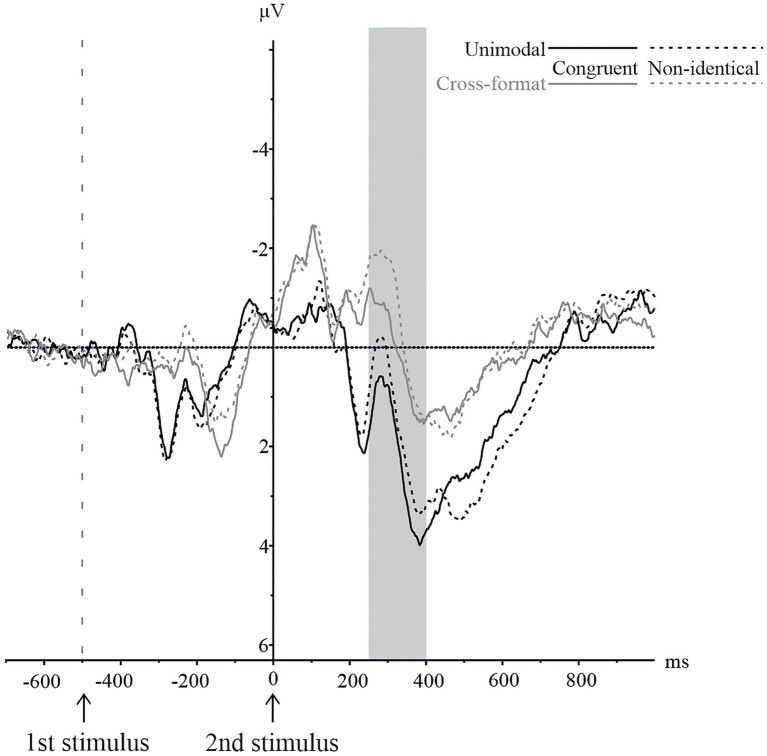
N400 component on the pooled central electrode cluster for numerically identical and non-identical number pairs in unimodal visual and cross-format auditory–visual blocks. Solid lines represent ERPs for identical, and dashed lines for non-identical number pairs. ERPs are shown in black for unimodal items and in grey for cross-format items.

Figure 4 provides the averaged waveforms of the central electrode cluster by identity and modality. As can be seen, the amplitudes of the waveforms were more negative for non-identical than identical number pairs in both unimodal and cross-format blocks. A 2 × 2 repeated measures ANOVA was conducted with identity (identical vs. non-identical) and modality (unimodal vs. cross-format) as within-subject factors. There was a significant main effect of identity, *F*(1,16)=11.19, *p*=0.004, $\eta_{p}^{2}$ =0.41, with more negative peak amplitudes for non-identical than identical number pairs. The main effect of modality was also significant, *F*(1,16)=11.51, *p*=0.004, $\eta_{p}^{2}$ =0.42. For the cross-format block, the peak amplitudes were more negative than for the unimodal block. The interaction identity x modality was not significant (*F*<1).

To ensure that the identity-based effect was present in both modality conditions, we conducted two separate ANOVAs with identity (identical vs. non-identical) as within-subject variable. For the unimodal block, there were more negative peak amplitudes for non-identical than identical number pairs, *F*(1,16)=7.11, *p*=0.017, $\eta_{p}^{2}$ =0.31. Similarly, in the cross-format block, there were more negative peak amplitudes for non-identical than identical number pairs, *F*(1,16)=7.75, *p*=0.013, $\eta_{p}^{2}$ =0.33.

### Discussion

The behavioral and electrophysiological results of Experiment 1 support the view that number words and digits are linked *via* number semantics when explicit numerical decisions are required. Behaviorally, participants were faster to judge whether the second number of a pair was larger or smaller than 5 if the previous number was numerically identical, pointing to a priming effect. Critically, this facilitation effect was found for both unimodal and cross-format number pairs.

Electrophysiologically, both the unimodal and the cross-format conditions elicited similar components. However, there were distinct effects of numerical identity in the unimodal and the cross-format conditions: Unimodal effects of numerical identity were found in the early time window between 100 and 200ms after stimulus onset, as well as in the later time window between 250 and 400ms. This suggests that pairs of visual-Arabic digits are linked at two different stages: First, both digits are rapidly and automatically integrated (as indexed by the N1 component), and second, their numerical content is processed semantically (as indexed by the N400 component). In contrast, cross-format effects were only found in the later time window between 250 and 400ms. This dissociation implies that while both conditions involved semantic processing of the number pairs, only the unimodal condition involved a rapid and automatic integration of both constituents of a number pair. In other words, our results show that cross-format integration of numerical content occurs less rapidly than within-format integration. Conversely, a previous study did report effects of numerical identity with pairs of non-symbolic quantities and visual-Arabic digits already in the N1 time window (Liu et al., 2018). Therefore, it could be reasoned that the integration of different forms of symbolic number (digits, number words) takes longer than the integration of non-symbolic and symbolic quantities. However, there are certain methodological pitfalls to consider: In the study by Liu et al. (2018), all participants performed a behavioral estimation task with hundreds of trials immediately before the passive ERP task. In this estimation task, they were asked to estimate the quantity of the non-symbolic stimuli and type in their answers as Arabic digits, thus possibly entailing subvocal rehearsal of the respective number words. As identical non-symbolic stimuli were utilized during the passive ERP task, this may have likely provoked the co-activation of the respective Arabic digits, as well as the verbalized number words during the task.

Our finding of a more pronounced N400 for numerically non-identical than identical number pairs is in line with previous numerical cognition studies investigating the ERP correlates of semantic incongruencies (e.g., Niedeggen et al., 1999; Szücs et al., 2007: Szücs and Soltész, 2010; Pinhas et al., 2014). The finding of an N400 effect of numerical identity in the cross-format condition supports the hypothesis that number words and digits are only indirectly linked *via* their underlying numerical meaning, as proposed by semantic models of transcoding (e.g., Power and Dal Martello, 1990; McCloskey, 1992). Thus, the ERP results strongly suggest that the constituents of a number pair are processed semantically.

We set out to investigate the link between digits and number words. In this experiment, we did not find any evidence for an early automatic cross-modal link. The presence of the later N400 effect of numerical identity for cross-format number pairs does suggest that representations of number words and digits were linked at a later time point. The N400 component is thought to reflect semantic processing; thus, our cross-format N400 effect suggests that number words and digits may be linked *via* number semantics. However, the task demands of Experiment 1 might have provoked an activation of semantic content, because participants were explicitly required to make numerical judgments. Arguably, participants may have actively and semantically processed the first number of a pair in order to facilitate the subsequent number judgment on the second number. This interpretation could also account for the behavioral priming effect we observed. It is also important to point out that our numerical judgment task involved response selection processes. This makes it difficult to distinguish whether the observed effects are due to numerical processing or response selection (Göbel et al., 2004).

In summary, we observed ERP effects of numerical identity for cross-format pairs of number words and visual-Arabic digits in Experiment 1. However, while unimodal pairs of visual-Arabic digits were associated with early N1 effects of numerical identity (pointing to an automatic integration), cross-format numerical identity effects only emerged in the later N400 time window (pointing to semantic processing). In order to disentangle whether these cross-format N400 effects were due to the nature of the numerical judgment task, we performed another experiment in which participants were not explicitly required to access the underlying magnitude representation.

In Experiment 1, we had difficulties in finding the most suitable baseline correction. We had to settle on a period rather far away (−700 to −500ms) from the onset of the target stimulus. As we can still expect amplitude changes due to the presentation of the first number after around 500ms, we decided to increase the stimulus onset asynchrony (SOA) by 500ms in Experiment 2.

## Experiment 2

### Participants

The sample comprised 19 healthy volunteers recruited at the University of Graz, Austria (age: *M*=25.2years, *SD*=3.1; 8 males and 11 females). One additional participant had to be excluded from the data analysis because of noisy data. All participants were native speakers of German and had normal or corrected-to-normal vision, as well as normal hearing status. Participants received course credit or 10€ for participation. The study complied with the Declaration of Helsinki and approval was obtained from the ethics committee of University of Graz. Participants provided written informed consent prior to participation.

We conducted a power analysis to determine sample size “pwr” package (Champely, 2020) in R (R Core Team, 2020). We set power to 0.80 and the probability of alpha error to 0.05, corresponding to the convention by Cohen (1988). To obtain a conservative estimate, we decided to consider the smallest effect size of numerical identity found in Experiment 1, $\eta_{p}^{2}$ =0.242. The power analysis revealed a minimum sample size of N=10. Thus, sufficient power is guaranteed for our current sample of N=19.

### Stimuli and Procedure

Participants were presented with numbers and letters, some of which were moving, while others were stationary. Participants were instructed to indicate the movement direction for moving numbers, but not for moving letters *via* keypress. Unknown to the participants, stimuli were organized into 192 standard trials and 36 filler items per block. Standard trials consisted of a number pair, followed by a number moving horizontally across the screen. In half of the standard trials, the two numbers of a pair were identical (both numbers had the same numerosity, e.g., 1–1) or non-identical (the numbers were numerically different, e.g., 1–9). This sums up to a total of 96 identical and 96 non-identical trials per block. Stimuli consisted of digits and number words corresponding to the numerosities 1, 4, 6 and 9.

Note that the overt task (response to moving numbers) did not require participants to actively access the magnitude of the numbers. Importantly, the EEG analysis (see below) focused on the second number of the number pair preceding the moving number and not on the moving number itself, which makes our task a passive paradigm with respect to analysis of numerical congruency. Moreover, this design ensured that processing of the number pairs was not contaminated by eye movement artifacts caused by the moving numbers.

Since participants only had to respond after every third item, we wanted to make sure that they also had to actively attend to the first two items. Therefore, we included 12 filler items in which moving numbers appeared at the very beginning or after the presentation of just one number. To make sure that participants not only react to the perception of movement they were only asked to respond to moving numbers and not letters. Thus, we inserted 24 filler items with moving letters instead of numbers, which participants were instructed not to respond to. While the focus of Experiment 2 was to analyze numerical identity, additional factors were controlled to avoid predictive learning: Each first number was followed with the same probability by either the same or one specific different number (e.g., 1–1, 1–9). Also, number pairs did not predict the subsequent moving number: The moving number either had the same numerosity as the preceding number (25% of cases) or not (75% of cases). In half of the moving numbers, the movement direction and numerical size (a larger number, ≥6, moves to the right side of the screen or a smaller number, ≤4, moves to the left side of the screen) matched. In the other half, they did not (a larger number, ≥6, moves to the left side of the screen or a smaller number, ≤4, moves to the right side of the screen).

There were two experimental blocks: The unimodal block consisted only of visually presented digits, whereas in the cross-format block, the first number was always a spoken number word, while the second number and the moving number were visual-Arabic digits. At the beginning of the experiment, participants completed 12 practice trials with feedback. In the middle and at the end of each experimental block, participants had the opportunity to take a break. The order of the two blocks was counterbalanced.

Visual-Arabic stimuli were presented in white on black background with a height of 4 degrees of visual angle at the center of the display. Similar to Experiment 1, we controlled for low-level perceptual adaptation effects. For that reason, visual-Arabic numbers were displayed in one of four slightly different spatial locations at one degree of visual angle from the center of the display. The location followed a pre-defined pseudorandom order, in which no two stimuli appeared at the same location. In each trial, visual-Arabic numbers immediately following each other were displayed in different spatial locations. Number words were presented by one of four speakers (two male and two female voices). All number words had a duration of 500ms.

**Figure 5:**
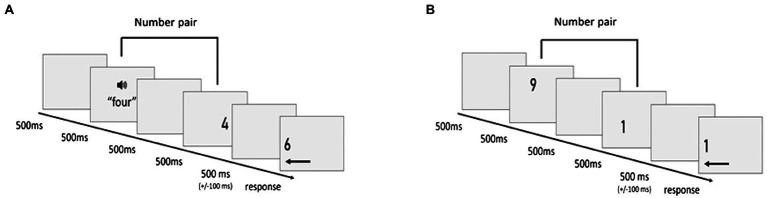
Examples of different trials of the ERP paradigm: **(A)** unimodal visual block, non-identical number pairs, **(B)** cross-format auditory–visual block, identical number pairs.

As illustrated in Figure 5, each standard trial proceeded in the following order: Blank screen (500ms), first number (500ms), blank screen (500ms), second number (500ms), and blank screen (jitter: 400–600ms). We analyzed ERPs in response to the second number. At the end of each trial, a digit moved horizontally to the left or the right side of the screen, until it stopped at a distance of four degrees of visual angle from the borders of the screen. The number moved at a constant speed of 1.67 pixels per frame. Participants were required to press the keyboard arrow corresponding to the direction of the movement either during the movement of the digit or after its arrival at the stationary position at the border of the screen. They were instructed to press the right arrow with their right index finger and the left arrow with their left index finger. If participants did not respond within 4s of stimulus onset, the next trial was presented.

### ERP Recording and Data Analysis

We employed the same ERP recording protocol as in Experiment 1, and the steps for preprocessing and data analysis were identical, except for baseline correction. The time window of −200 to 0ms before onset of the second number of a pair served as the basis for baseline correction. Only segments with a correct response were considered. All participants had at least 74 valid segments in each of the four conditions; thus, all participants were included in the analyses. For the unimodal block, an average of 93.26 (*SD*=3.87) identical and 92.32 (*SD*=3.84) non-identical segments were retained. For the cross-format block, we kept 92.63 identical (*SD*=5.35) and 92.26 non-identical (*SD*=5.24) segments.

### Results

#### Behavioral Measures: Accuracy

To ensure that participants attended to the presented stimuli, they were required to respond to moving numbers, but not letters. On average, participants correctly reacted to 99.32% of the moving numbers (*SD*=0.48%; range: 98.53–100.00%), while they incorrectly responded to only 9.58% of letters (*SD*=6.63%, accuracy range: 65.96–97.87%). This high response accuracy suggests that participants were attentive toward the presented stimuli.

#### N1

**Figure 6:**
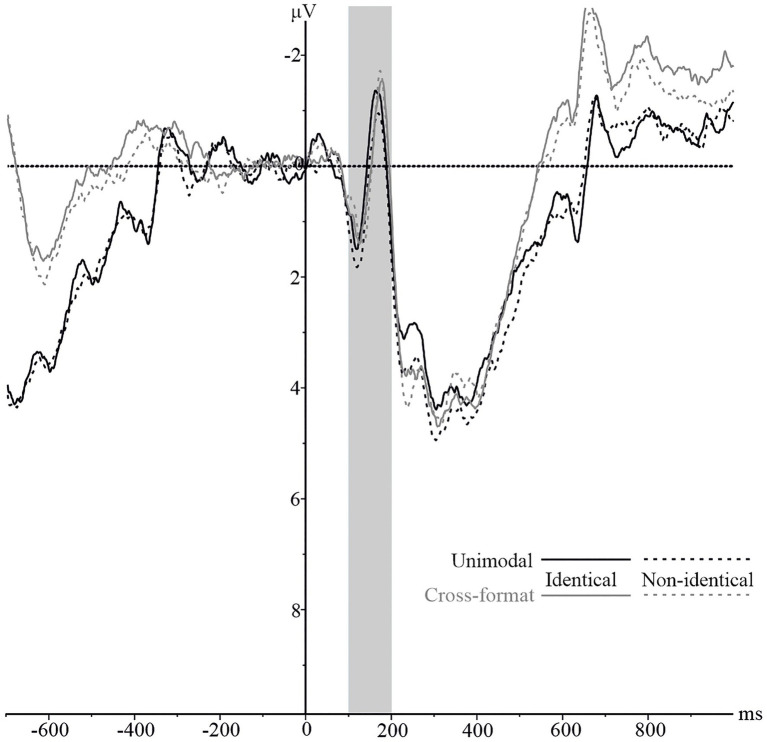
N1 component on the pooled parietal electrode cluster for numerically identical and non-identical number pairs in unimodal visual and cross-format auditory–visual blocks. Solid lines represent ERPs for identical, and dashed lines for non-identical number pairs. ERPs are shown in black for unimodal items and in grey for cross-format items.

As illustrated in Figure 6, the averaged waveforms of the parietal electrode cluster were more negative for non-identical than identical number pairs in the unimodal block, whereas this was not the case for the cross-format block. We performed an identity x modality ANOVA which showed that both main effects were not significant: modality, *F*(1,18)=4.02, *p*=0.060, $\eta_{p}^{2}$ =0.18 and identity, *F*(1,18)=1.53, *p*=0.231, $\eta_{p}^{2}$ =0.08. However, there was a significant interaction of modality x identity, *F*(1,18)=4.89, *p*=0.040, $\eta_{p}^{2}$ =0.21.

To follow up on the significant interaction, we conducted two separate repeated measures ANOVAs with identity (identical vs. non-identical) as within-subject factor. These revealed a significant effect of identity for the unimodal block, *F*(1,18)=5.42, *p*=0.032, $\eta_{p}^{2}$ =0.23, with more negative peak amplitudes for identical than non-identical number pairs. For the cross-format block, there was no significant difference, *F*(1,18)=0.03, *p*=0.864, $\eta_{p}^{2}$ =0.00.

#### N400

**Figure 7:**
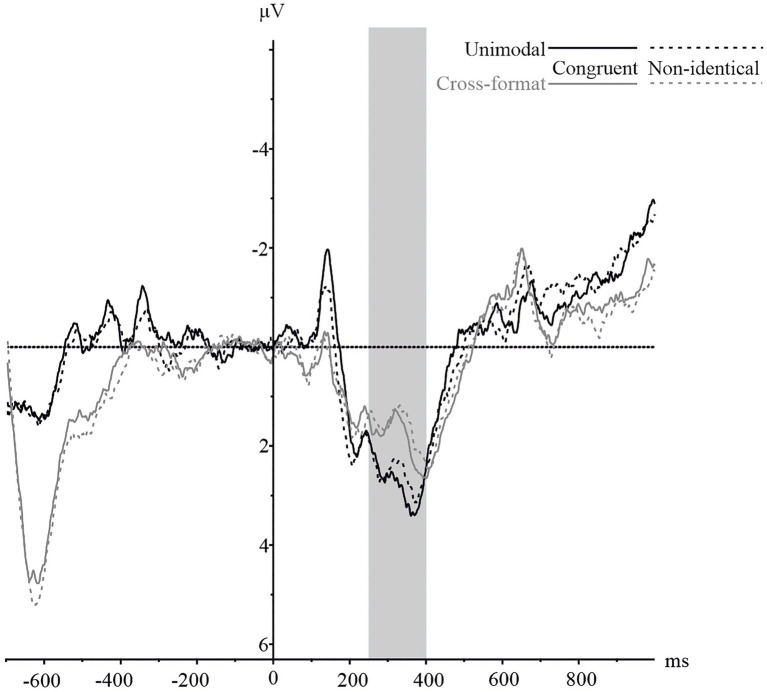
N400 component on the pooled central electrode cluster for numerically identical and non-identical number pairs in unimodal visual and cross-format auditory–visual blocks. Solid lines represent ERPs for identical, and dashed lines for non-identical number pairs. ERPs are shown in black for unimodal items and in grey for cross-format items.

The averaged waveforms of the central electrode clusters by numerical identity and experimental block are depicted in Figure 7. As illustrated in Figure 7, the averaged waveforms for non-identical number pairs were more negative than for identical number pairs, especially in the unimodal block. However, an identity x modality ANOVA showed no significant main effect of identity, *F*(1,18)=0.78, *p*=0.388, $\eta_{p}^{2}$ =0.04. There was a significant main effect of modality, *F*(1,18)=9.95, *p*=0.005, $\eta_{p}^{2}$ =0.36, with more negative peak amplitudes in the cross-format than in the unimodal block. The interaction identity x modality was not significant, *F*(1,18)=0.443, *p*=0.514, $\eta_{p}^{2}$ =0.024.

Because the sample size was relatively small, we also conducted a Bayes factor (BF) analysis to determine the relative strength of the alternative hypothesis compared to the null hypothesis for the N400 ERP peak amplitude data (Dienes, 2014; Wagenmakers et al., 2018). We used the JASP software version 0.14.1.0 (JASP Team, 2020). As our repeated measures ANOVA contained several factors, we calculated inclusion Bayes factors (BF_Inclusion_), which can be interpreted as evidence in the data for including a predictor (Wagenmakers et al., 2018). We found extreme evidence for the main effect of modality, BF_Inclusion_=153.41. For the main effect of identity, we found evidence for the null hypothesis, BF_Inclusion_=0.30. For the interaction, we found no evidence for the alternate hypothesis, BF_Inclusion_=0.36.

### Discussion

The results of Experiment 2 support the notion that number pairs and digits are not automatically linked when no numerical judgments are involved. While presenting both a condition with unimodal pairs of visual-Arabic digits and cross-format pairs of number words and digits similar to Experiment 1, the present task was designed to be passive and to not require semantic number activation.

Unimodally, we found an early N1 effect of numerical identity and some traces of an N400 effect. Although the cross-format condition elicited similar components, these were not affected by numerical identity. The dissociation in the N1 component points again to an automatic integration of unimodal pairs of digits, but not of cross-format pairs of digits and number words. Unimodal integration of numerical stimuli therefore appears to happen automatically and involuntarily, even in a task not requiring any link between both constituents of a number pair. This supports the suggestion that processing of numerical identity is not limited to situations in which numerical information is explicitly processed (Liu et al., 2018). However, this automatic integration does not appear to extend beyond the visual modality, as we did not find any evidence for automatic integration of numerical information from different sensory modalities. This corroborates findings from a previous study on visual–auditory integration of number words and non-symbolic quantities in preschoolers, which also did not report any early signs of integration (Pinhas et al., 2014).

The N400 effect which we found in Experiment 1 was basically eliminated by employing a task in which semantic activation of the underlying magnitude was not provoked. This is in contrast to the study by Pinhas et al. (2014), which reported higher N400 amplitudes for numerically non-identical than identical pairs of visually presented non-symbolic quantities and spoken number words. Arguably, the link between number words and their non-symbolic counterparts might be tighter than between number words and digits, at least in children. Further studies are necessary before drawing the conclusion that there is no automatic link between number words and digits in the absence of semantic processing.

## General Discussion

We investigated the possibly automatic link between number words and digits and examined whether unimodal numerical identity is associated with different ERP effects compared to cross-format numerical identity. We were interested in two ERP components: the early N1 component which is associated with automatic processing (Liu et al., 2018) and the later N400 component which is associated with the semantic processing of numbers (Niedeggen et al., 1999; Galfano et al., 2004; Paulsen and Neville, 2008; Szücs and Soltész, 2010).

We found parietal N1 ERP effects of numerical identity in the early time window from 100 to 200 ms after stimulus onset. However, this was only found for unimodal, but not cross-format number pairs. On the one hand, this implies that visual-Arabic digits are rapidly and automatically linked, even if numerical processing is not actively required. On the other hand, automatic integration does not appear to extend to cross-format pairs of digits and number words. This suggests that cross-format integration of numerical information from different symbolic formats occurs less rapidly than within-format integration. This contrasts with previous research on the cross-format integration of visually presented non-symbolic and symbolic numerosities, which appear to be automatically linked (Liu et al., 2018). Therefore, symbolic numbers and their non-symbolic counterparts may have a tighter link than different symbolic representations.

Moreover, it is possible that our finding of an automatic integration of two visual-Arabic digits is partly due to perceptual visual similarity (c.f. Cohen, 2009) seeing that numerically identical number pairs showed a greater visual overlap than numerically non-identical ones. Evidence from an fMRI adaptation study with visual-Arabic and Chinese numerals suggests that brain responses to numerical stimuli are not only based on the numerical meaning but are also influenced by perceptual overlap (Holloway et al., 2013). Nonetheless, we did take measures to decrease this influence by varying the spatial locations and fonts of pairs of visual-Arabic digits. We found unimodal and cross-format N400 effects of numerical identity, but only when the active task required numerical decisions. As the N400 component is believed to reflect semantic processing (Kutas and Federmeier, 2011), it can be deduced that a semantic link between two numbers is not established directly, but instead individuals actively have to access the underlying meaning. While we did not find any evidence for an automatic link between cross-format number pairs as indexed by the N1 component, we did find an ERP effect of numerical identity for cross-format number pairs in the later N400 window.

Crucially, this was only true when an activation of semantic content was provoked by task demands (Experiment 1). When semantic activation was not provoked by the task (Experiment 2), we could not observe any N400 effects, neither unimodally nor in the cross-format condition. This suggests that number words and digits are indirectly linked *via* their underlying numerical magnitude. However, it is important to note that the SOA between the constituents of a number pair varied between Experiment 1 and Experiment 2: While the SOA was 500ms in Experiment 1, it was 1,000ms in Experiment 2. Therefore, it is important to consider the possibility that this longer SOA caused semantic priming to fade away and dissipate (e.g., Xiao and Yamauchi, 2017). First evidence suggests that this may indeed be the case in the domain of numerical processing: Lin and Göbel (2019) conducted a behavioral study in which participants were asked to indicate whether cross-format pairs of visual-Arabic digits and auditory number words with varying SOAs (−500ms to +500ms) were identical or not. Lin and Göbel (2019) observed cross-format numerical distance effects (indicating semantic processing) across all SOAs, but these distance effects decreased with increasing SOAs. Therefore, it is possible that the lack of semantic priming we observed in Experiment 2 (with an SOA of 1,000ms) may have been related to using a long SOA. However, the precise effect of SOA length on semantic priming is still a matter of debate: Other studies suggest that semantic priming of lexical content is facilitated by longer SOAs (i.e., longer than 500ms, e.g., Chen and Spence, 2018; Roelke et al., 2018). If this were also the case for cross-format number pairs, the lack of semantic priming we observed in Experiment 2 may not have been observed because, but perhaps rather in spite of a long SOA.

As discussed above, the current study found no evidence for a direct link between visual-Arabic numbers and number words. These results can easily be integrated with semantic models of transcoding, as they suggest accessing the other form through semantic activation. Asemantic models, however, are based on the assumption that transcoding takes place in the absence of semantic activation. Indeed, there is neuropsychological evidence supporting the view that semantic activation is not mandatory for transcoding: Dehaene and Cohen, 1995 described the case of a patient with Gerstmann’s syndrome who was selectively impaired in tasks requiring access to the number semantics but showed intact transcoding skills. Nonetheless, the precise cognitive mechanisms linking number words and digits remain as yet unclear. A crucial step in support of asemantic models would be to demonstrate the existence of an automatic integration of number words and digits. The current study was, however, unable to do so in healthy adults.

An analogy for the absence of an automatic link between symbolic representations of number (i.e., number words and digits) can be found in the neighboring domain of reading. As a cautionary note, it is important to mention that there are distinctive differences between reading and number processing: While numbers are inherently meaningful as they reflect non-symbolic quantities, letters often have to be grouped to strings to form meaningful words. However, there are interesting parallels, as both letters and digits are culturally acquired symbols: While we communicate about quantities with number words and digits, our script code consists of strings of letters or characters that are used to reflect speech sounds. Evidence from cross-script priming suggests that there is indeed no automatic link between different scripts within the same language (Okano et al., 2013). Specifically, cross-script priming can be investigated in languages containing pairs of symbols that map onto the same phonological representation. For instance, Japanese has two syllabaries, Hiragana and Katakana, which both have characters directly corresponding to the same Japanese syllables (e.g., Hiragana さ and Katakana サ both represent/sa/). Similar to our behavioral findings on the cross-format priming of number words and digits, substantial behavioral priming effects for primes that are displayed in different scripts from their targets have been reported both in lexical decision (Pylkkänen and Okano, 2010) and semantic categorization tasks (Okano et al., 2013). However, ERP-based findings suggested that cross-script prime-target pairs are not automatically linked, but rather *via* their underlying semantics as indexed by the N400 component (Okano et al., 2013).

Interestingly, similar to cross-script priming, there is a body of evidence supporting the notion of an automatic link between non-symbolic quantities and their symbolic counterparts (Galfano et al., 2004; Paulsen and Neville, 2008; Pinhas et al., 2014; Liu et al., 2018). It would be fruitful to disentangle possibly different mechanisms supporting the integration of non-symbolic visual quantities and number words, compared to symbolic Arabic digits and number words. A future challenge for numerical cognition research therefore is to investigate whether the automatic integration of non-symbolic quantities and their symbolic counterparts contributes to higher-order skills such as mental calculation.

It is important to acknowledge some limitations of the current study. One might argue that the observed absence of an automatic and asemantic integration of auditory number words and visual-Arabic digits may be partially due to our experimental design. First, early ERP components such as the N1 effect are known to be modality-dependent (Donohue et al., 2011), and second, the length of the SOAs between cross-format number pairs may have impacted the automatic association (Lin and Göbel, 2019).

Concerning modality-dependence, it is possible that the observed N1 effects of numerical identity constitute a unique feature of unimodal processing of visually presented numbers. This may also explain why Liu et al. (2018) observed early ERP effects for the integration of visually presented quantities and digits, while the present study could not find any early signs of cross-format integration between auditory number words and visual-Arabic digits. However, we compared ERPs within and not across modalities. Indeed, for both unimodal and cross-format conditions, we compared the N1 effect evoked by the presentation of the second number of a pair, which was always presented visually (i.e., unimodal: two visual-Arabic digits; cross-format: one auditory number word followed by one visual-Arabic digit). Since no cross-format comparisons were made, the observed similarities and differences should not stem from modality-specific effects.

A central parameter in early cross-modal integration is the temporal proximity of the stimuli (e.g., Donohue et al., 2011). The shorter the SOA, the stronger the observed priming effect (Lin and Göbel, 2019). Short SOAs, however, do not enable the separate analysis of ERPs, as high amplitude components of the prime stimulus may still take place during the time window of the target stimulus. To keep the priming effect as large as possible while keeping the ERPs of the first and second stimuli as dissociated as possible, we employed a 500-ms delay in Experiment 1 and a 1,000-ms delay in Experiment 2. We managed to observe unimodal identity effects with such a design and assume that if there were automatic markers of cross-modal integration, we should have been able to detect those with our design. However, while we used different SOAs in Experiments 1 and 2, our results cannot directly inform the question about the effect of manipulating SOAs because of additional design differences between the two experiments. Such effects should be examined in future experiments only manipulating SOAs.

## Conclusion

Our findings contribute to the debate on the nature of the integration of different symbolic number forms (visual-Arabic digits and auditory number words). In both experiments, unimodal pairs of visual-Arabic digits were consistently found to be automatically integrated across both experiments, but we did not find any evidence for an early and automatic cross-format integration. In our experiments, evidence of the cross-format association between visual-Arabic digits and verbal number words emerged late and involved semantic activation. The present study thus does not support the notion of an automatic and asemantic cross-format integration of number words and visual-Arabic digits in adults.

## Data Availability Statement

The data collected for this study is publicly available on the Open Science Framework and can be accessed at: https://osf.io/p7ksn/.

## Ethics Statement

The studies involving human participants were reviewed and approved by Ethics committee of the University of Graz. The patients/participants provided their written informed consent to participate in this study.

## Author Contributions

KL and SG developed the project concept. SF, FK, CB, FC, AS, and SG developed ideas for the design of the experiments. SF programmed the experiments, and analyzed and interpreted the data together with FK. SF, CP-S, CB, and AS acquired the data, and IP provided the required resources. SF drafted the manuscript under the supervision of FK and KL, and all authors provided critical revisions. All authors approved the final version of the manuscript for submission.

## Funding

This work was supported by the FWF, Austria (grant no. I 2778-G16) and the ESRC, UK (grant no. ES/N014677/1).

## Conflict of Interest

The authors declare that the research was conducted in the absence of any commercial or financial relationships that could be construed as a potential conflict of interest.

## Publisher’s Note

All claims expressed in this article are solely those of the authors and do not necessarily represent those of their affiliated organizations, or those of the publisher, the editors and the reviewers. Any product that may be evaluated in this article, or claim that may be made by its manufacturer, is not guaranteed or endorsed by the publisher.

## Footnotes

^1^While the first number of the country codes reflect continental information (e.g., Zone 1: North and Central America, Zones 3–4: Europe), the full country codes do not represent topographical organization. Some neighboring countries, like Sweden and Norway, do have neighboring codes, but others do not.

^2^This was also qualified by numerical distance, but as described below, numerical distance cannot be tested in our study.
